# A Modular Geometrical Framework for Modelling the Force-Contraction Profile of Vacuum-Powered Soft Actuators

**DOI:** 10.3389/frobt.2021.606938

**Published:** 2021-03-03

**Authors:** Samuel Dutra Gollob, Clara Park, Bon Ho Brandon Koo, Ellen T. Roche

**Affiliations:** ^1^ Department of Mechanical Engineering, Massachusetts Institute of Technology, Cambridge, MA, United States; ^2^ Institute for Medical Engineering and Science, Massachusetts Institute of Technology, Cambridge, MA, United States

**Keywords:** soft robotics, numerical model, artificial muscle, virtual work, vacuum-powered soft actuator

## Abstract

In this paper, we present a generalized modeling tool for predicting the output force profile of vacuum-powered soft actuators using a simplified geometrical approach and the principle of virtual work. Previous work has derived analytical formulas to model the force-contraction profile of specific actuators. To enhance the versatility and the efficiency of the modelling process we propose a generalized numerical algorithm based purely on geometrical inputs, which can be tailored to the desired actuator, to estimate its force-contraction profile quickly and for any combination of varying geometrical parameters. We identify a class of linearly contracting vacuum actuators that consists of a polymeric skin guided by a rigid skeleton and apply our model to two such actuators-vacuum bellows and Fluid-driven Origami-inspired Artificial Muscles-to demonstrate the versatility of our model. We perform experiments to validate that our model can predict the force profile of the actuators using its geometric principles, modularly combined with design-specific external adjustment factors. Our framework can be used as a versatile design tool that allows users to perform parametric studies and rapidly and efficiently tune actuator dimensions to produce a force-contraction profile to meet their needs, and as a pre-screening tool to obviate the need for multiple rounds of time-intensive actuator fabrication and testing.

## Introduction

Soft robotics is a growing field, owing somewhat to an increasing demand for machines that can interact more safely with humans and their environment, generate complex multi-degree-of-freedom motions, and resist impact damage (Rus and Tolley, 2015). The sub-field of soft artificial muscles is relevant, as they are commonly used to actuate soft robots as opposed to traditional motors. Although a variety of artificial muscle actuation schemes have been developed, including shape-memory alloys (Kim et al., 2009), tension cables (Calisti et al., 2011), and phase transitions (Miriyev et al., 2017), fluidic actuation is widely used, as it is compatible with soft matrices, with programmed fluidic channels, and provides a means to increase actuator volume and effective stiffness analogous with the contraction and stiffening of biological muscle (Rus and Tolley, 2015). Pneumatic artificial muscles have been used in, and theorized for, a range of applications, from medical implantable devices (Roche et al., 2017; Mac Murray et al., 2018), to exoskeletons (Polygerinos, et al., 2015a; Porter et al., 2020), and both soft and rigid robotic applications (Andrikopoulos et al., 2011).

Most soft pneumatic actuators described in the literature operate with positive pressure, often involving a section of the actuator which expands with pressure and a strain-limiting component which guides the elastic expansion in a desired direction. This duality has been achieved by creating geometrical asymmetry in elastomeric actuators (Ogura et al., 2009; Katzschmann et al., 2016), introducing an off-axis strain-limiting material for bending motions (Martinez et al., 2012; Mosadegh et al., 2014), and reinforcing the outer skin of the actuator with fibers (Chou and Hannaford, 1996; Connolly et al., 2015; Deimel and Brock, 2016; Wirekoh and Park, 2017). Although positive pressure actuators can produce complex motions and large forces (Rus and Tolley, 2015), they have limited contraction ratios, high actuation pressure requirements, and are subject to delamination or bursting (Chou and Hannaford, 1996; Sanan et al., 2014; Niiyama et al., 2015; Li et al., 2017). Owing to their dependence on volume increase for contraction, they pose a design challenge for applications where space is constrained.

Vacuum-operated soft pneumatic actuators are an alternative to positive pressure actuators that can avoid some of these pitfalls, while still achieving similar bending (Robertson and Paik, 2018; Tawk, 2018), linear (Yang et al., 2016; Yang et al., 2017; Li et al., 2017; Li et al., 2019; Felt et al., 2018; Lee and Rodrigue, 2019), and complex programmed motions (Li et al., 2017; Jiao et al., 2019). This class of actuators rely on a decreasing volume for actuation, in contrast to positive pressure actuators where the volume typically increases upon actuation. Similar to the strain-limiting operating principle for positive pressure actuators, vacuum actuators often involve a thin strain-limited “skin” that is responsible for a decrease in volume upon actuation, and a “skeleton” that limits compression to guide the volume decrease in a desired direction (Li et al., 2017; Felt et al., 2018; Tawk et al., 2018; Lee and Rodrigue, 2019). For the purpose of this paper, this type of vacuum actuator will be referred to as a “skin-skeleton vacuum actuator.” Particularly, this work focuses on skin-skeleton actuators that undergo linear contraction upon actuation. This class of actuators has achieved contraction ratios near or above 90%, is often lightweight, fast-moving, and resistant over many cycles, requires low actuation pressures, and produces a high power to weight ratio compared to positive pressure actuators (Li et al., 2017; Felt et al., 2018; Tawk et al., 2018; Lee and Rodrigue, 2019). As a result of these design features, these actuators have potential benefit for a variety of applications, especially those requiring large linear displacement which is challenging to achieve with commonly reported artificial muscles.

Previous work has developed a variety of actuator models, often based on the Finite Element Method (FEM), for describing their actuator designs (Agarwal et al., 2017; du Pasquier et al., 2019; Nguyen and Zhang, 2020; Polygerinos et al., 2015b). These models allow in-depth characterization of corresponding actuators, predicting buckling modes, stress distributions and and actuator motion as a function of pressure, expected force output, and cycle lifetime. FEM approaches have been shown to characterize actuators for their use in a particular application, and maintain the versatility of their design for other applications – for example Nguyen and Zhang (2020) characterize a family of modular cells using FEM that can be combined for curling, linear, and twisting motions as desired by the end user.

Though FEM models are successful in describing actuator performance in detail, their complexity means they are not ideal for higher-level design iterations and selection of broad design spaces for performance constraints. To our knowledge, a generalized, versatile model that can rapidly generate information on an actuator’s output is missing in the existing body of work. Such a model could be used before the time-consuming prototyping, material testing, and FEM model creation that comes with a more developed design.

Inspired by previous literature, we developed one such model that makes use of the virtual work principle to extract an actuator’s force-contraction output force based on its volume loss rate. This is implemented via a simple and versatile numerical algorithm using solely geometrical features of the actuator. The model can then overlay other components of the actuator–such as a restoring force–to better approximate its force output.

The force-contraction profile (FCP) is a common characterization metric to describe the actuator output force over the course of its contraction assuming a constant pressure, and it is nonlinear for most vacuum actuators, creating demand for tools that can allow one to understand and predict the FCP for a given actuator design. There have been a variety of simplified models that attempt to predict such profiles. While some models make use of force balancing analytical and numerical finite element models (Polygerinos et al., 2015b; Li et al., 2017), others have modelled actuator outputs using analytical solutions to the principle of virtual work (Chou and Hannaford, 1996; Li et al., 2017; Felt et al., 2018; Lee and Rodrigue, 2019), which allows a force profile to be estimated solely from the actuator’s geometry:

$$F=P*\frac{\text{d}V}{\text{d}s}$$ (1)

Where V is the actuator’s internal volume, P is the actuation pressure (usually assumed constant), and s is the contraction or current length of the actuator. Assuming no energy loss and an inextensible skin, the output of the virtual work equation (Eq. 1) can be used to estimate the force output of the actuator directly (Chou and Hannaford, 1996; Li et al., 2017). The models based on the principle of virtual work mentioned above apply an analytical solution derived from the design and geometry of the actuator in question, following a typical workflow: a skin geometry is defined, used to derive a formula for volume as a function of contraction, and the volume formula is differentiated. In one instance, this analytical approach was combined with a minimizing function (Felt et al., 2018) to allow for the skin to change in cross-sectional geometry to mimic the physical tendency to minimize volume in a vacuum.

**FIGURE 1:**
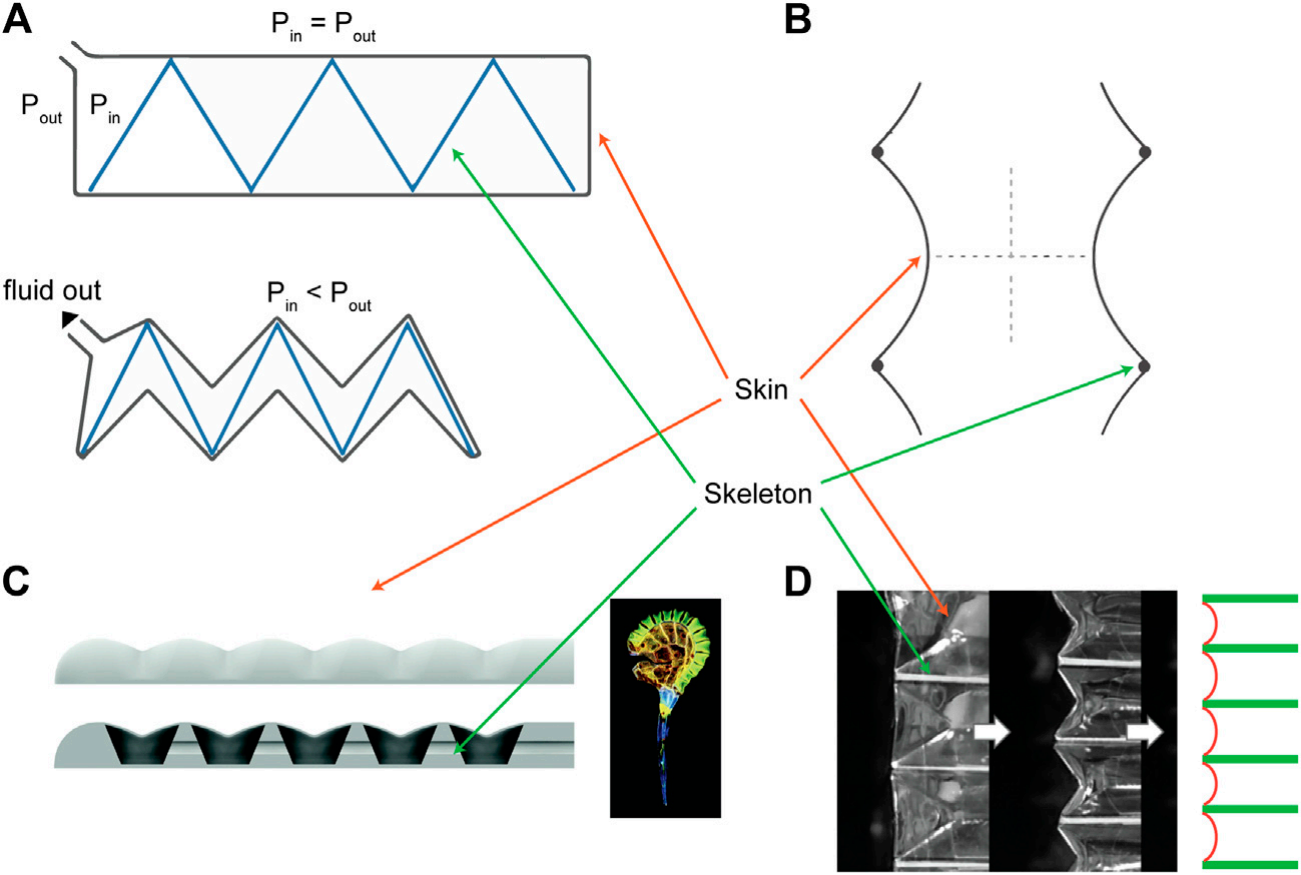
Overview of existing skin-skeleton vacuum actuator designs, highlighting the skin and skeleton components. These include **(A)** FOAMs (Li et al., 2017), **(B)** Bellows actuators (Felt et al., 2018), **(C)** bending soft actuators (Tawk et al., 2018), and **(D)** Origami bellows actuator (Lee and Rodrigue, 2019). All figure reproductions approved by publishers.

In this paper, we expand the use of the virtual work concept and present a generalized platform that enables rapid prediction of the FCP of a linearly contracting skin-skeleton vacuum actuator for any skin or skeleton geometry. By implementing a generalized numerical approach in MATLAB (MathWorks), we create a versatile model that can easily be applied to different actuator designs, without the need for the development of a separate analytical model for each design. To demonstrate the application and capabilities of the framework, we use it to model the FCPs of two representative types of linear skin-skeleton actuators: the bellows actuators (Felt et al., 2018; Figure 1A), and the Fluid-driven Origami-inspired Artificial Muscles (FOAMs) (Li et al., 2017; Figure 1B). The bellows actuator was chosen for its simple design and pre-existing modelling work, and the FOAM was chosen because its semi-rigid zigzag shaped skeleton adds geometrical complexity and behaves like a spring, adding an additional component to test the framework’s modularity. Finally, we validate the framework by experimentally characterizing actuators with varying geometric parameters. This framework has potential utility as a design tool for soft roboticists, or device designers, enhancing the computational efficiency of the virtual work principle with modularity, allowing rapid application to various actuator designs and geometries.

## Materials and Methods

### Conceptual Framework

**FIGURE 2:**
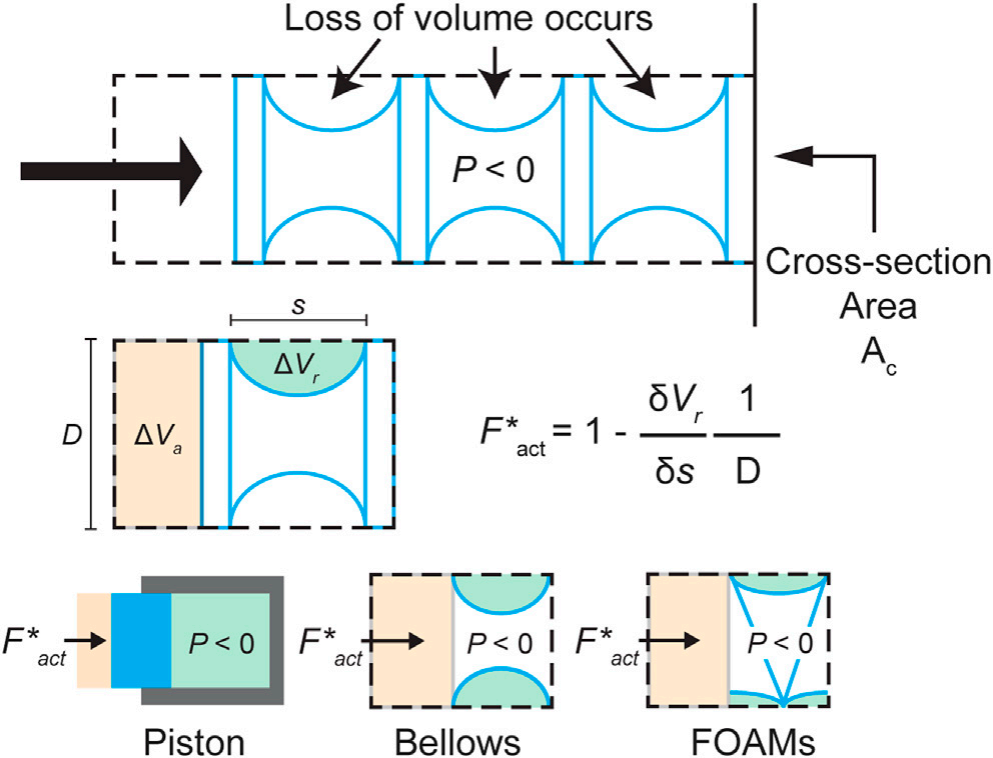
A schematic of the general skin-skeleton vacuum actuator working principle, conceptualizing the components for the model derivation, with relevant volume losses labeled. Bottom row shows how Eq. 2 can be applied to different actuator designs. ΔVr = volume loss in radial direction. $\Delta V_{a}$ = volume loss in axial direction. Ac = characteristic radial length. $F_{\text{act}}^{*}$ = scaled actuator output force. P = actuation pressure. s = current actuator length. D  = cross-sectional area.

Guided by the virtual work approach described in previous studies (Chou and Hannaford, 1996; Felt et al., 2018), we derived a simple generalized formula for the FCP of a vacuum actuator based purely on its geometry. As Figure 1 illustrates, most soft vacuum actuators with linear contraction motion exhibit volume loss in both the axial ($\Delta V_{a}$) and radial ($\Delta V_{r}$) directions. It is beneficial to categorize the volume loss in this way because, while the spatial derivative of the axial volume loss $\left(\text{d}V_{a}/\text{d}s\right)$ leads to a constant force profile, the derivative of the radial volume loss is responsible for the nonlinearity in the actuator FCP, as will be shown. This also allows for an easy non-dimensional transformation of the output, as can be seen in the brief derivation below, based on the labeled $V_{r}$ and $V_{a}$ values from Figure 2:

$$\begin{array}{l} V_{T\text{,act}}=A_{c}s-\;V_{r};\\ V_{T,\text{piston}}=A_{c}s, \end{array}$$

$$\begin{array}{l} \frac{F_{act}}{P}=\left(A_{c}-\frac{\text{d}V_{r}}{\text{d}s}\right);\;\\ \frac{F_{\text{piston}}}{P}=A_{c}, \end{array}$$

$$\frac{F_{\text{act}}}{F_{\text{piston}}}=F_{\text{act}}^{*}=1-\frac{1}{D}\left(\frac{dV_{r}}{ds}\right)\;,$$ (2)

where $V_{T}$ is the total internal volume of the actuator’s contractile cell, F is its output force in Newtons, P is the constant actuation pressure and D is a characteristic length that replaces the cross-sectional area $A_{c}$ in the case of a 2D simplification of the actuator. The subscripts act and piston refer respectively to the actuator in question and a piston of equivalent cross-sectional area, where a piston is defined as having no radial volume loss (Figure 2). $F_{\text{act}}^{*}$ is the piston-scaled force of the actuator, a non-dimensional force or a ratio of the actuator’s force output compared to that of its equivalent piston. In this case, the actuator is simplified as a two-dimensional equivalent, so $A_{c}$ becomes a characteristic radial length, D, while $V_{r}$ becomes a two-dimensional slice of the lost radial volume. As Eq. 2 shows, the scaled output force of the actuator is a function of the derivative of the radial volume loss over its contraction s. This assumes a constant pressure and constant bounding cross-sectional area (D). By setting the characteristic length D to 1, Eq. 2 describes a generalized scale-independent force profile curve.

As Figure 2 illustrates, this concept of radial volume can be translated into different actuator types. The goal of our framework is to allow for a generalized application of the radial volume rate concept, such that the force profile for different actuators can be extrapolated.

### Implementation into Model

**FIGURE 3:**
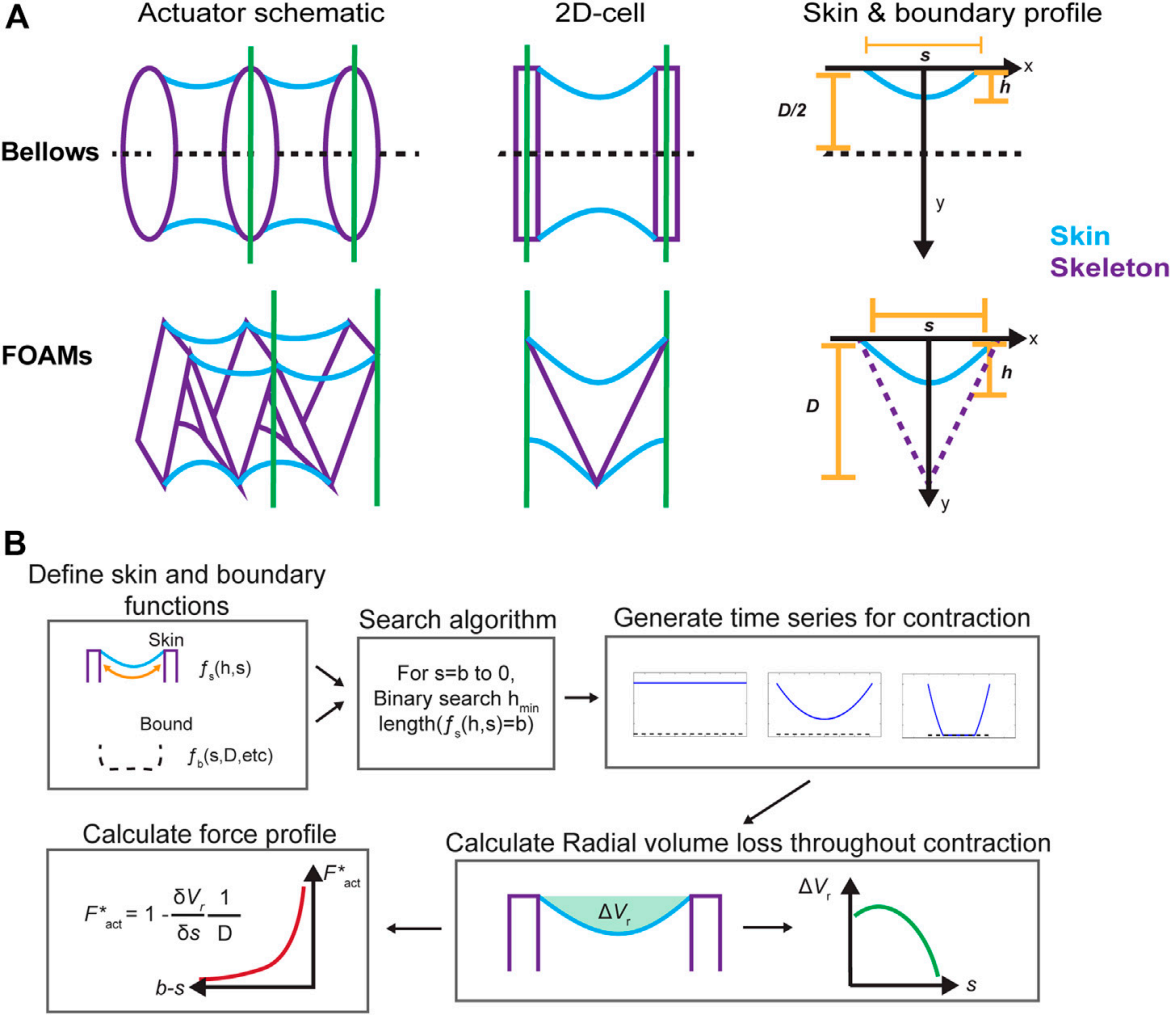
**(A)** Simplifying actuator geometry for virtual work model. **(B)** A schematic of model implementation. s = length of a contractile cell. h = skin sagging depth. D = actuator cross-sectional area. b = initial spacing between two contractile cells or constant length of skin section. $V_{r}$ = radial volume loss. $F_{s}$ = skin function. $f_{b}$ = boundary function. $F_{act}^{*}$ = piston-scaled actuator output force.

To generalize the concept of radial volume loss, we identify two components that can be used to represent a vacuum actuator in our model: a skin profile and a boundary profile. As Figure 3A demonstrates, the model requires that a vacuum actuator be discretized into contractile cells, similar to that used in the derivation. This cell is simplified as a two-dimensional shape with a zero-thickness skin, described by a function $f_{s}$, and a set of boundaries, described by the boundary function $f_{b}$.

In this paper, we focus on modelling the bellows (Felt et al., 2018) and FOAM (Li et al., 2017) actuators, because both fall into the skin-skeleton category, have linear motion, and have been well-described experimentally and analytically in the literature. The bellows actuator has a simple working principle due to the low number of components in its assembly and its minimal skeleton design, while the FOAM actuator is interesting for the resistance of the folded skeleton resistance to contraction, which imparts an opposing spring force to the actuator. In both cases, the characteristic skin function is a parabola of constant length fixed at both ends of the cell, chosen to emulate the profile of the actuator skin as it conforms to the underlying skeleton during contraction.

$$f_{s}\left(s,h\right)\left(x\right)=\;-h\left(1-\frac{x^{2}}{{\left(s/2\right)}^{2}}\right);\;\;-\frac{s}{2}\leq x\leq \frac{s}{2},$$ (3)

Where s is the length of the contractile cell, h is the skin’s “sag depth” into the actuator, and x is the axial coordinate of a point along the skin (Figure 3A shows these variables in a schematic). The algorithm can accept alternative skin functions, provided they include the input h for sag depth, as discussed in the model implementation section.

The boundary conditions were defined separately for the bellows and FOAM actuators, as shown in Figure 3A. Although there are no defining structural bounds for the bellows actuator skin, a boundary at the midline was defined since the axisymmetry of the actuator causes the skin to contact itself on actuation for cases where the gap distance between rings is greater than one diameter (Felt et al., 2018). For the FOAM, the boundary was defined by the zigzag shaped skeleton, which was assumed to have zero thickness. The boundary equations are as follows:

$$f_{b,\text{bellows}}\left(x\right)=\;-\frac{D}{2},$$ (4)

$$f_{b,\text{FOAM}}\left(L,s\right)\left(x\right)=\frac{2d}{s}\left|x\right|-d;\;\;d=\sqrt{l_{i}^{2}-{\left(\frac{s}{2}\right)}^{2}},\;$$ (5)

where L is the constant length of one of the sections of the zigzag of the FOAM’s skeleton, defined as $D÷\cos\left(\theta_{i}/2\right)$, and d is the height of the skeleton for a given value of s. Figure 3A includes a schematic of the skin and boundary functions.

With the specified skin and boundary functions, the FCP can be solved, as illustrated in Figure 3B. The major computational section of the model is the process for calculating the geometrical configuration of the skin for each point in the contraction (between contractile cells, equal to the skin length, b, to 0). This is achieved via a binary search algorithm, which attempts to find the lowest value of h that leads to a skin configuration of length b (equal to the initial gap length of b), as the model assumes an inextensible skin. The algorithm requires a function $l_{s}\left(h\right)$, which outputs the length of the skin configuration for a given sag depth h. For a given increment i in the contraction, the binary search starts at $h_{i,1}=h_{i-1,\text{final}}$ (or 0 for the first increment), and adds a constant step value to h, updating the lower bound $h_{i,\text{lower}}$ until it reaches a value of h that returns a length greater than the desired target, at which point it sets the upper bound $h_{i,\text{upper}}$. The next increment, $h_{i,\text{next}}$ is linearly interpolated between $h_{i,\text{lower}}$ and $h_{i,\text{upper}}$ based on how far from the target section length the from the upper and lower bound configurations are:

$$h_{i,\text{next}}\;=\frac{\left(h_{i,\text{lower}}dist_{\text{upper}}\;+\;h_{i,\text{upper}}dist_{\text{lower}}\right)}{dist_{\text{upper}}+dist_{\text{lower}}},$$ (6)

where $dist_{j}=\left|l_{s}\left(h_{i,j}\;\right)-b\right|$. If $h_{i,\text{next}}$ is below the target, it becomes $h_{i,\text{lower}}$, and similarly for $h_{i,\text{upper}}$ in the case that the increment is above the target value. Eventually, this converts to the target value within a tolerance, but to return the smallest valid h value, the function returns only once the upper and lower bounds are close to each other by a certain tolerance. This assumes the section length is monotonically increasing as a function of h, but can be adjusted to account for local maxima/minima using traditional optimization function methods.

The final crucial piece of the algorithm is the skin profile function, which solves the geometrical configuration of the skin for a given h. Given a value for h, it first calculates the skin profile (in this case, always a parabola) and identifies any intercepts with the boundary function. If there is no intercept, it returns the skin profile as a 2D-array of x-y coordinates. If there is an intercept with the boundary, the function draws the section of the parabola up to the intercept, then draws the section of the boundary for the remainder of the way or until the second intercept. The process is repeated recursively to complete a profile.

The result of the binary search function and the skin profile function working in unison is a skin configuration for each contraction increment i, from s at full to zero length, referred to as the “time series” in Figure 3B. This represents the shape of the actuator’s skin throughout the contraction. If, at some increment, the skin profile can no longer be solved, as is the case for the bellows actuators once the skin is in full contact with the boundary, that increment defines the end of the contraction. The $V_{r}$ value for each increment is calculated and then numerically differentiated following the function for $F_{\text{act}}^{*}$ to finally generate the FCP.

### Finite Element Modeling of FOAM Actuators

In the case that an actuator has a non-negligible spring force that resists its contraction, we hypothesized that one can super-impose the calculated spring resistance force with the virtual work model FCP to reach an accurate estimate of the actuator’s true FCP. To estimate the spring resistance force from skeleton, a quasi-static FEM model of a FOAM skeleton was created in Abaqus/Explicit (Dassault Systèmes). The skeleton was modeled with a 30-degree fold angle, 50 × 20 × 10 mm bounding dimensions, and 1 mm thickness and modelled as a linearly elastic polyvinyl chloride plastic (Density = 1.4 g/cc, Young’s Modulus = 2.4 GPa, Poisson’s Ratio = 0.3 as defined by the manufacturer specifications) and 8-node linear brick, reduced integration, hourglass control (C3D8R) elements. This skeleton was fixed at one end, restricting both rotation and displacement, and a displacement boundary condition of 35 mm was applied to the other end, compressing the skeleton gradually over time. The reaction force of the skeleton in the axial direction was extracted to quantify its spring resistance. In parallel, a full FOAM model was generated, where the same skeleton was surrounded by a bounding skin. The skin was modelled using a thermoplastic elastomer with high stiffness (Density = 0.8 g/cc, Young’s Modulus = 600 MPa, Poisson’s ratio = 0.3, average properties from MatWeb) with 4-node, quadrilateral, stress/displacement shell elements (S4R) of thickness 0.02 mm, with a membrane idealization (such that the skin is dominated by tensile forces). A general, frictionless contact interaction was defined for all elements in the simulation. The skin was fixed at one end and a variable displacement condition was applied to another end (no boundary conditions were applied to the skeleton). Then, the model was run with the following actuation steps. First, a preload of vacuum pressure up to −70 kPa was applied inside the skin while the two ends of the actuator were held fixed. Once the pressure reached −70 kPa, the force was held constant and a gradual displacement boundary condition of 25 mm was applied to one end of the skin, while the other was kept fixed, allowing the actuator to contract. The axial reaction force at the fixed end of the skin was then extracted to quantify the FCP of the FOAM actuator. For the analysis, we compared the FCP generated by the FOAM actuator in the FEM and the net force predicted by the virtual work model, which was calculated as the pure FCP from the geometrical virtual work model subtracted by the skeleton spring force obtained in the FEM.

### Actuator Fabrication

#### FOAM Actuators

**FIGURE 4:**
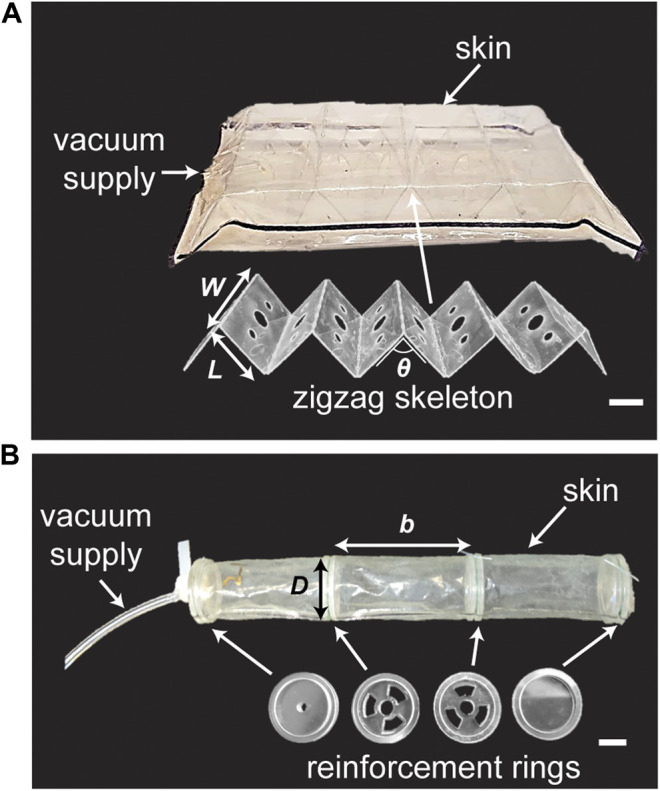
Actuator prototypes. **(A)** A FOAM actuator contains a linear zigzag skeleton with multiple segments (W x L) separated by *θ* inside a bag of thin skin. W = width of skeleton, L = length of a skeleton section, *θ* = skeleton initial angle. **(B)** A vacuum bellows actuator contains multiple reinforcement rings equally spaced by R inside a thin tubular skin. R = ring spacing. D = ring diameter. Scale bar = 1°cm.

FOAM actuators consist of a thin skin layer surrounding a rigid zigzag structure that serves as the skeleton (Figure 4A). For the skin layer, we sealed two sheets of 0.05 mm-thick thermoplastic elastomer (Fibreglast) using an impulse sealer for 20 s along two edges at a nominal spacing of 53 mm. For the skeleton, 0.254 mm-thick polyester sheet (McMaster-Carr) was laser cut in a series of 10 segments (L = 20 mm, W = 40 mm) with minor features on each segment to allow air flow and perforated lines between the segments to help folding. The skeleton was manually folded along the perforated lines at desired angles of 30, 60, and 90 degrees. For assembly, the skeleton was inserted into the skin membrane and sealed using an impulse sealer at skeleton lengths of 60, 110, and 150 mm, respectively. A piece of PTFE was used to create a gap in the seal for subsequent tube insertion. To ensure that the skeleton material did not slide inside the skin during actuation, we used thermoformable anchors at the ends of the skeleton that were sealed with the skin, and therefore fixed at each end.

#### Vacuum Bellows

A vacuum bellows actuator consists of a thin tubular membrane surrounding rigid rings that are evenly spaced along the axis of the tube (Figure 4B). Two 0.04 mm polyethylene sheets (McMaster-Carr, 7889T28) were sealed along two edges at a nominal width of 40 mm for 4 s using an impulse sealer (Hacona, H-6705) to make a 25 mm-diameter tubular membrane. For the rigid rings, a three-part assembly consisting of one concentric ring surrounded by two thinner annular rings placed at the edges of the inner ring was fabricated. The inner ring was made of 4.76 mm acrylic (McMaster-Carr) and laser cut to form an outer diameter of 40 mm with minor cut features that enable airflow between segments for middle segments and a 3.175 mm center hole for placing the tubing at one end. The outer annular rings were made from 1.59 mm acrylic with an inner diameter of 20 mm and outer diameter of 25 mm, and were bonded to the inner ring using cyanoacrylate (Loctite). The assembled rigid rings were positioned inside the membrane and orthogonally to the wall, and then secured around the groove created in the ring assembly using fishing line (9442T2, McMaster-Carr). The remainder of the ring assemblies were positioned along the membrane at the desired spacing and fastened in similar way. A 3.175 mm OD polyurethane tube was inserted through the first ring assembly for vacuum supply and the ends of the membrane were sealed to the acrylic using cyanoacrylate adhesive and SilPoxy (Smooth-On).

### Testing

#### Actuator Testing

To obtain FCPs for each actuator, we measured the force-displacement curve using a mechanical tensile tester (Instron 5944) for all actuators. The actuators were held at the ends with a 2-kN load cell (Supplementary Figure S1) and allowed to contract at a rate of 100 mm/min until the force reached zero. Constant vacuum pressures of −15 kPa for the vacuum bellows and −25 kPa for the FOAM actuators were applied throughout the test using a manual vacuum gauge (IRV10-N07, SMC). Actuation pressure was measured using a TruWave pressure sensor (Edwards Lifesciences) and the average pressure for each actuator was used for normalizing the measured output forces. For both Bellows and FOAM actuators, three replicates (n = 3) were used for each experiment (i.e n = 3 for each value of R for Bellows, each value of θ for FOAMs).

#### Skin Material Testing

To compare the mechanical properties of different skin materials, a uniaxial tensile test was performed on an Instron 5944 at a rate of 1 mm/min. All rectangular test specimens had widths of 20 mm and lengths of 40 mm. The thickness of the skin materials was 0.04 mm for the polyethylene film and 0.05 mm for the thermoplastic elastomer. The corresponding Young’s modulus was obtained by taking the best fit slope between strains of 0% to 5%, and the average value (n = 5) was used for scaling the forces in the model.

#### Spring Compression Testing

To characterize the spring force generated by the skeletons in FOAM actuators, we measured the force exerted by a 30-degree zigzag skeleton during compressive loading at a rate of 100 mm/min on a mechanical tensile tester (Instron 5944). The spring constant was derived by taking the slope of force-displacement graph and the mean value (n = 3) was used to model the spring force for the FOAM actuators.

## Results

### Bellows

#### Force Profile Explanation

**FIGURE 5:**
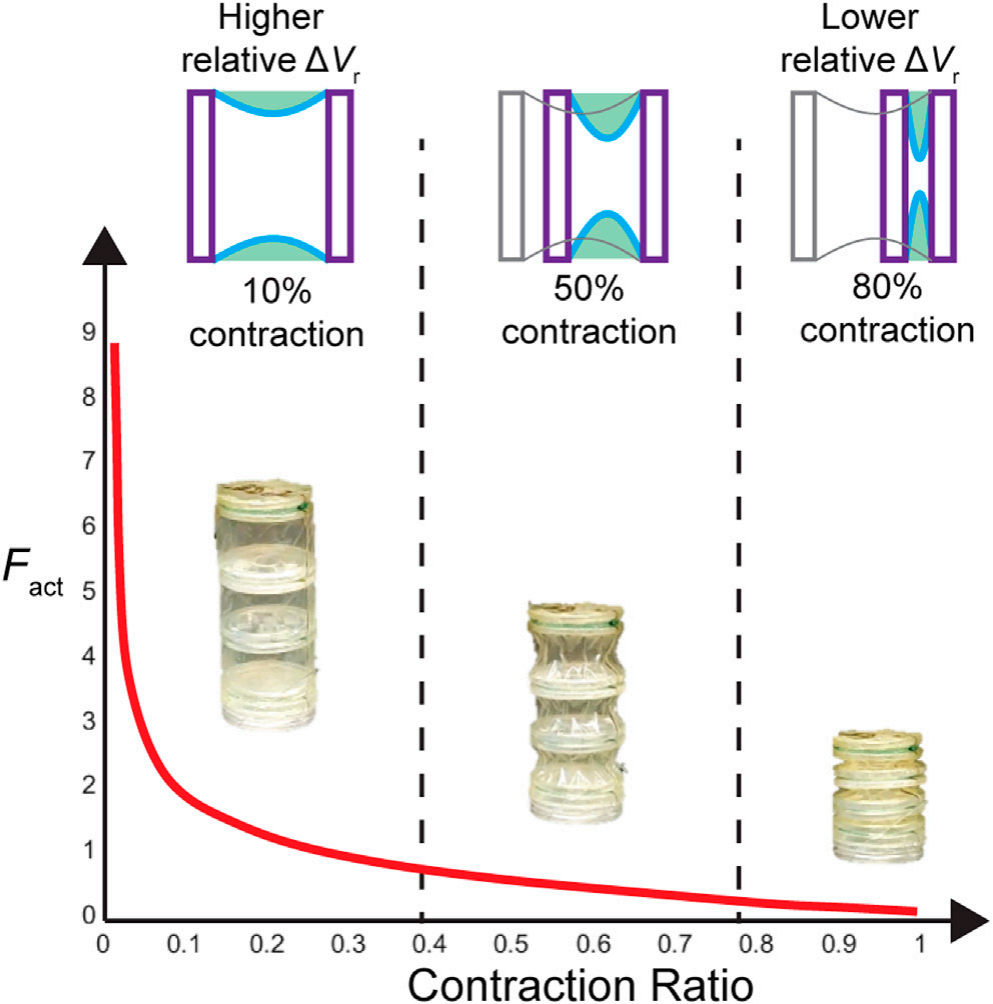
Illustration of the force-contraction profile of bellows actuators, depicting the geometrical reasoning around the shape of the profile based on the concept of the virtual work function. Initially, the actuator skin is completely straight, and as it contracts, the parabolic profile leads to a rapid loss in radial volume, which justifies the large initial force value (large derivative). As the contraction nears its middle and end, the parabolic skin profile sags less and becomes narrower, leading to a smaller rate of volume loss and thus a smaller piston-scaled force.

Figure 5 illustrates the shape of a force profile for a bellows actuator, overlaid with a simplified time series of the skin profiles generated by the model. As shown, the large force output at the start of the contraction is directly tied to the large relative change in radial volume at the beginning, as the skin experiences the greatest drop. In later stages, the output force curve flattens as the skin gets closer to its final configuration, and the loss in radial volume decreases.

#### Parameter Sweep

**FIGURE 6:**
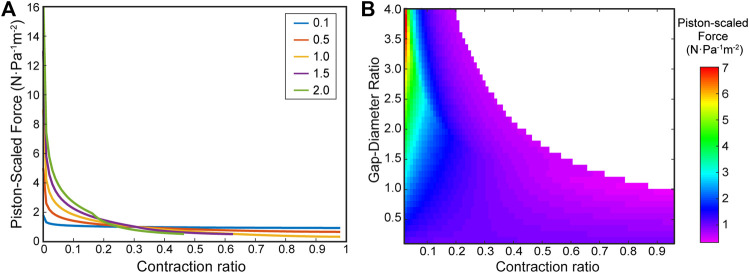
Virtual work model for vacuum bellows actuator with varying ring distances. **(A)** Force-contraction profile for varying gap-diameter ratios (R) = 0.1, 0.5, 1, 1.5, 2, showing the trends caused by varying ring gap. **(B)** A heatmap from a high-resolution parameter sweep of the gap-diameter ratio. The color bar indicates piston-scaled force predicted by the model, and the contraction ratio is cut off at 0.04 for the purposes of visualizing the contrast throughout the heatmap (large magnitudes past 0.04 lead to colors focused on high values).

We performed a parametric sweep of different ring gap-diameter ratios using our virtual work model to evaluate the effect of the ring spacing on the force profile of bellows actuators. Figure 6A shows the calculated force profiles for varying ring gap-diameter ratios, R=b/D. The peak force decreases with decreasing gap distance, the force profile becomes more linear with decreasing gap distance, and gap distances greater than one diameter in length lead to a maximum scaled contraction equal to $R^{-1}$. Figure 6B overlays the full parameter sweep of values of R from 0 to 4, demonstrating how much stroke and force can be generated by an ideal vacuum bellows actuator for the given ring and gap dimensions.

#### Experimental Validation

**FIGURE 7:**
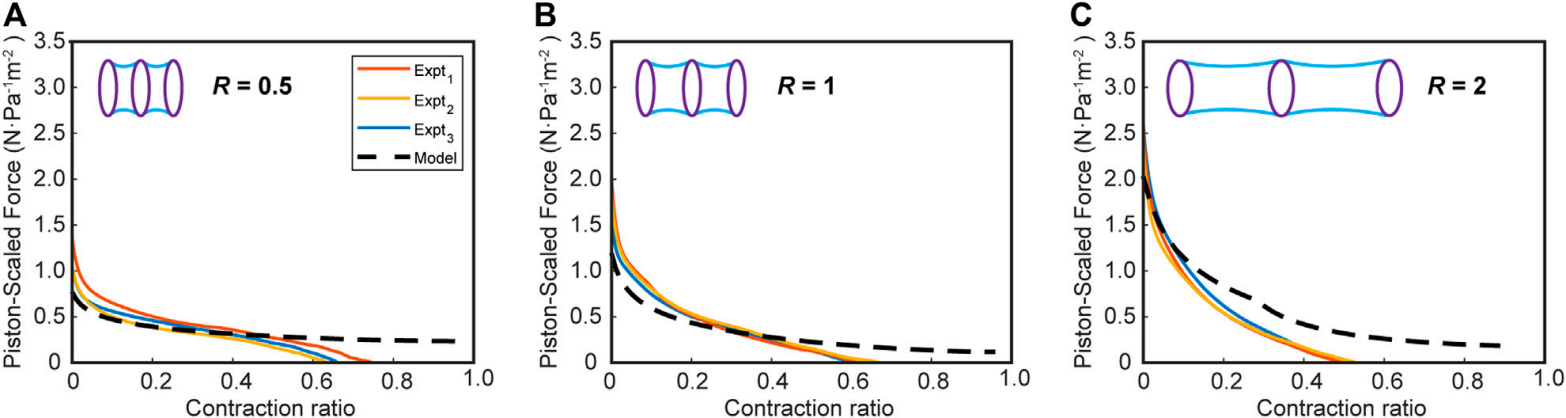
The force-contraction profile for vacuum bellows actuators in the experimental and scaled virtual work model for varying ring gap-diameter ratios **(A)** R = 0.5, **(B)** R = 1, **(C)** R = 2 for a ring diameter of 25 mm. Number of experiments, n = 3.

To validate the model experimentally, we measured the force-contraction behavior of bellows actuators with varying gap ratios, R, of 0.5, 1, and 2. Figure 7 shows the piston-scaled force produced by the actuator over the scaled contraction (defined as displacement divided by the actuator length). As a comparison, the virtual work model FCP with the equivalent R is overlaid with the results, after a magnitude scaling factor is applied. This factor accounts for the material properties of the actuator skin, which has some extensibility compared to the inextensible skin assumption in the model. We derived a relationship for the scaling factor through a set of FEM experiments extracting the FCP of a bellows actuator with varying skin thicknesses and stiffnesses, from which we could derive an empirical relationship between output force and Young’s modulus (E) and skin thickness (t) (See Supplementary Figure S3). We found the scaling factor is a function of ${\left(Et\right)}^{1/3}$, which agrees with Roark’s formula for the tension in a cable with a distributed load (Young and Budynas, 2002)–see Supplementary Section 1.1 for further details. Li et al., (2017) observed the relevance of skin Young’s modulus and thickness for the output of vacuum actuators, which informed our decision to develop this scaling factor. For our bellows actuators, which use a 0.04 mm thick polyethylene film as the skin material (E = 127 MPa), we scaled the output force predicted by our model by 0.35, calculated through our derived scaling factor equation (see Supplementary Eq. S1).

### FOAMs

Next, we expanded our virtual work model framework to the FOAMs actuators developed by Li et al., (2017). These actuators not only require a different boundary geometry, but the addition of a spring force from the folded rigid skeleton.

#### Parameter Sweep

**FIGURE 8:**
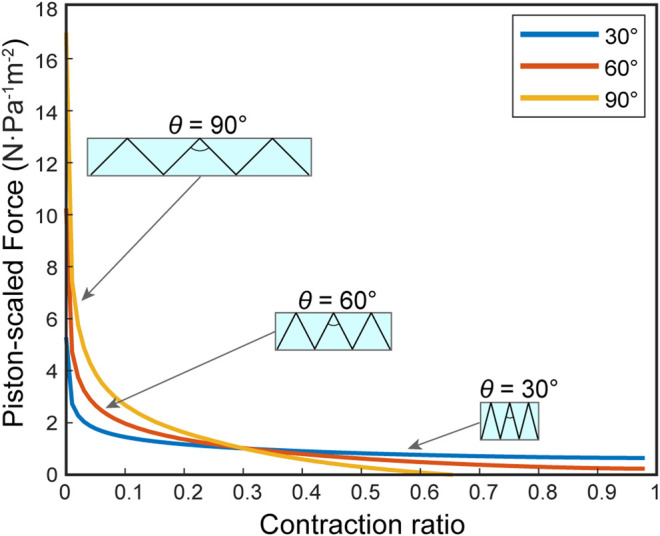
The virtual work model prediction of force-contraction profile for FOAM actuators with skeleton angles (*θ*) = 30, 60, and 90 degrees. The profiles are based only on the geometrical features, without the inclusion of spring force.

First, we predict the FCP of the FOAMs geometry without an additional spring factor, only considering the effect of its triangular bounding function shape. The skeleton angle *θ* in FOAM actuators is analogous to the ring gap distance R in bellows actuators, as the fold-to-fold distance in the zigzag skeleton is a function of θ: R=2⁡tan(θ/2). Figure 8 demonstrates the FCPs predicted from the FOAMs boundary setup for varying *θ* in absence of spring force.

#### Finite Element Modeling of FOAM Actuators

**FIGURE 9:**
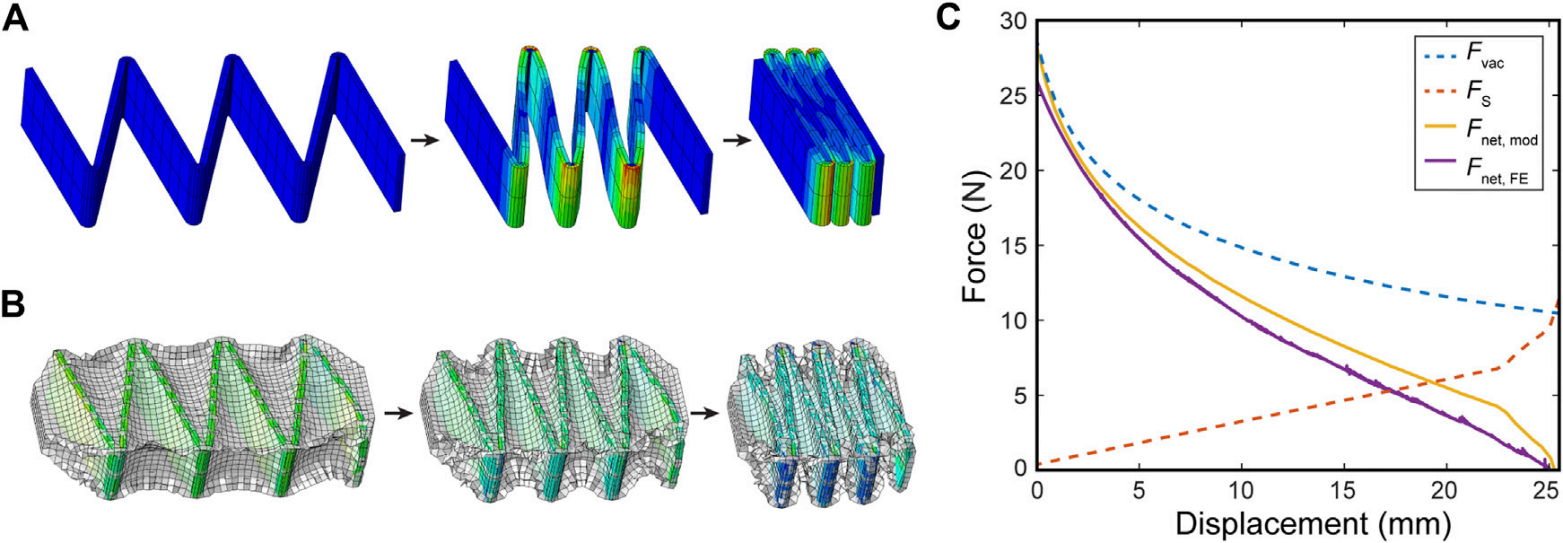
**(A)** Deformation of a 30-deg skeleton during compression test in a Finite Element (FE) model. **(B)** Free contraction of FOAM actuator containing the same skeleton under constant negative pressure in a FE setup. **(C)** A comparison of FE Model and virtual work model for FOAM actuators for θ = 30, P = −70 kPa. Fs = Skeleton spring force predicted by FE. $F_{\text{vac}}$ = Force predicted by virtual work model without spring force. $F_{\text{net},\text{mod}}$ = $F_{\text{vac}}\;–\;F_{s}$ = Net force predicted by the MATLAB model. $F_{\text{net},FE}$ = FOAM force predicted by FE.

Figure 9A shows deformation of skeleton during spring compression test performed in FEM, and Figure 9B shows contraction of a FOAM actuator using the same skeleton under constant pressure. Figure 9C compares the FCPs generated with the FEM (Fnet,FE) and virtual work models (Fnet,mod), demonstrating that the virtual work model can accurately describe a force profile when the skeleton spring reaction force is subtracted from it. The peak force from the virtual work model was within a 7% error and the full contraction length was within a 1% error.

### Actuator Selection Example

To demonstrate the use of this model as a design guidance tool with its large parameter sweep capabilities, we developed an example for selecting the ideal actuator gap ratio (R) for a simplified exoskeleton or humanoid robot arm application using a bellows actuator. As shown in Figure 11A, the actuator is anchored across an elbow and must be able to counter a constant torque equivalent to 1 N applied at the end of the arm $\left(F_{\text{res}}\right)$, throughout the bending of the arm $\left(\alpha :\left[\frac{\pi }{16},\frac{5\pi }{6}\right]\right)$. The forearm anchor $d_{1}$ is close to the elbow and the shoulder anchor $d_{2}$ is further from the elbow to mimic the layout of a bicep. The biomechanical analysis with a $d_{1}$ of 5 cm, $d_{2}$ of 20 cm, and $d_{3}$ of 30 cm shows the required contraction is 36% and the peak force coincides with the zero-contraction point, which agrees with the intrinsic FCP of a bellows actuator. The optimized actuator for this case is one with maximum peak initial force and a contraction of at least 36%. Given that R is proportional to peak force and inversely proportional to contraction (Figure 6B), the optimized problem is solved by identifying the highest R value with a final contraction of at least 36%. A simple maximizing algorithm was applied with these conditions to the parameter sweep data, and an optimized R of 2.4 was found.

Finally, by choosing a scaling factor of 0.5 (to match the TPU above, a common polymer skin material) and actuator cross section of 5 × 5 cm (as a realistic dimension for this application), the actuator output curve was compared to the required output, as overlaid in Figure 11B, and a linear pressure-force relationship is used to derive the necessary actuation pressure throughout a quasi-static contraction. The same was repeated for an R of one to illustrate how the pressure control conditions change with actuator selection.

## Discussion

### Bellows Actuator Model and Experimental Comparison

In our work, the bellows actuator was chosen as the first application as its simple design allows it to be realistically idealized by the assumptions made in the model, without the need for additional components. In Figure 6, we demonstrate that the model can predict FCPs with varying gaps as well as performing large parameter sweeps with high granularity. Our findings are consistent with the major trends found in the analytical model from Felt et al., (2018). Namely by increasing R, the force magnitude on the initial part of the contraction is increased while the total stroke is decreased for an R above 1. Additionally, the smaller the value of R the more constant the FCP becomes. The heat map in Figure 6B also agrees closely with that in Felt et al., (2018) and can give users a holistic view of the design space and help inform the actuator selection process (see Supplementary Figure S8 and Section *Actuator Selection Example*). As can be seen in Supplementary Figure S8, the models are in close agreement–the estimated contraction lengths, the trend of force distribution are similar, though the Felt model implemented a different more complex skin profile function which is a strong possible reason for the discrepancy in scaled force magnitudes.

Comparing the model and experimental results in Figure 7, we observe a close agreement in terms of FCP. During the early phase of the contraction, particularly for the R = 0.5 and one sets, there is very close agreement but we see some deviation of the modeling results from the experimental results toward the end of contraction in all cases for bellows actuators (Figure 7), most likely due to the zero-thickness skin and zero-energy loss assumption in the virtual work model. With these assumptions, the skin collapses in an orderly fashion until the cell contraction reaches 100%, when in reality the thickness and chaotic crumpling of the skin causes a nonlinear restoring force that increases toward the end of the contraction. This phenomenon of a nonlinear decreasing force is corroborated in the experimental results from Felt et al., (2018).

To demonstrate that this skin restoring force is a significant contributor to the discrepancy between the model and experimental results, we performed a second experiment where the actuators were compressed with an internal gauge pressure of zero and their restoring force was measured (see the Supplementary Material for experiment details). By overlaying this restoring force with the model for individual actuators, the output curve is estimated more closely (Supplementary Figure S5). The FCP curves downward at the end of the contraction, matching the experiment, and the final contraction is more closely approximated (with an average error of 14%). Given the complex mechanics of this crumpling skin restoring force, future work would be needed to create a predictable model based on this phenomenon. The effect of a restoring spring force is further investigated in our FOAM model, where the skeleton’s restoring force is characterized by a linear trend.

### FOAMs Model and Experimental Comparison

Comparing the FOAMs parameter sweep figure (Figure 8) with the results see in Figure 6, we can see the same general trend is preserved, where there are higher forces and lower maximum contraction with increasing *θ*. One key difference is demonstrated by the *θ* = 90° case, where the maximum contraction is restricted when the scaled force magnitude reaches zero, meaning the radial volume loss derivative reaches a maximum, unlike for the bellows actuator where maximum contraction is bounded by a geometrical constraint when the skin comes into maximum contact with the boundary. This demonstrates the importance of defining specific boundary functions in the virtual work model, based on actuator design.

As hypothesized, Figure 9 illustrates the importance of including the skeleton spring force in the model for predicting the FOAM force profile. Though the initial FCP from the pure virtual work model matches that of the FEM, once the spring force becomes non-negligible, the virtual work model deviates and predicts much higher force and contraction at the end of the FCP. The results also show that the spring force can be subtracted linearly as a post-processing step, rather than having to be integrated into the virtual work model framework itself.

**FIGURE 10:**
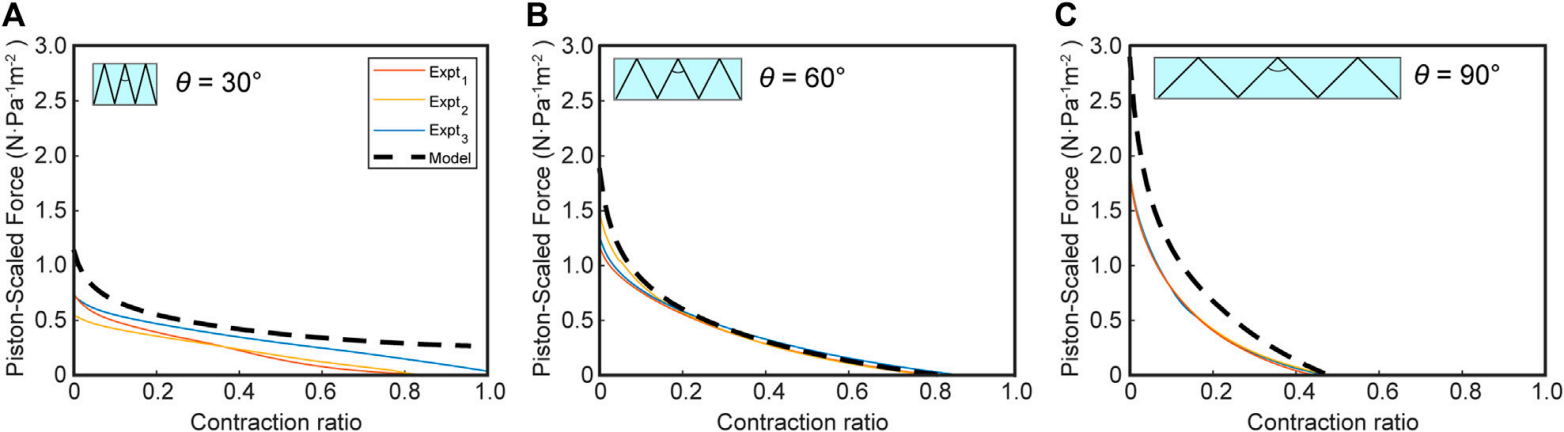
Force-contraction profile for FOAM actuators in experimental and virtual work model for varying skeleton angles [*θ*]; **(A)** 30°, **(B)** 60°, **(C)** 90°. Model = predicted net force = (pressure-only force without skeleton)-(skeleton spring force). Number of experiments, n = 3.

In keeping with this demonstration, the experimental results in Figure 10 show that the virtual work model with the subtracted skeleton spring force can closely predict the FOAM actuator force profile. The results of excluding a spring force in the model are two-fold; an over-estimated contraction distance and a larger force profile toward the end section, as is visualized in Figure 9. In the case of *θ* = 30°, the contraction is smaller compared to our prediction. This indicates that the spring resistance force from the skeleton may contribute less when the absolute displacement of the skeleton is smaller (because the 30-degree skeleton is comparatively shorter than the others). In this case, similar to the bellows actuator, the loss may be dominated by resistance from the skin, which is not captured in this model.

### Actuator Selection Example

**FIGURE 11:**
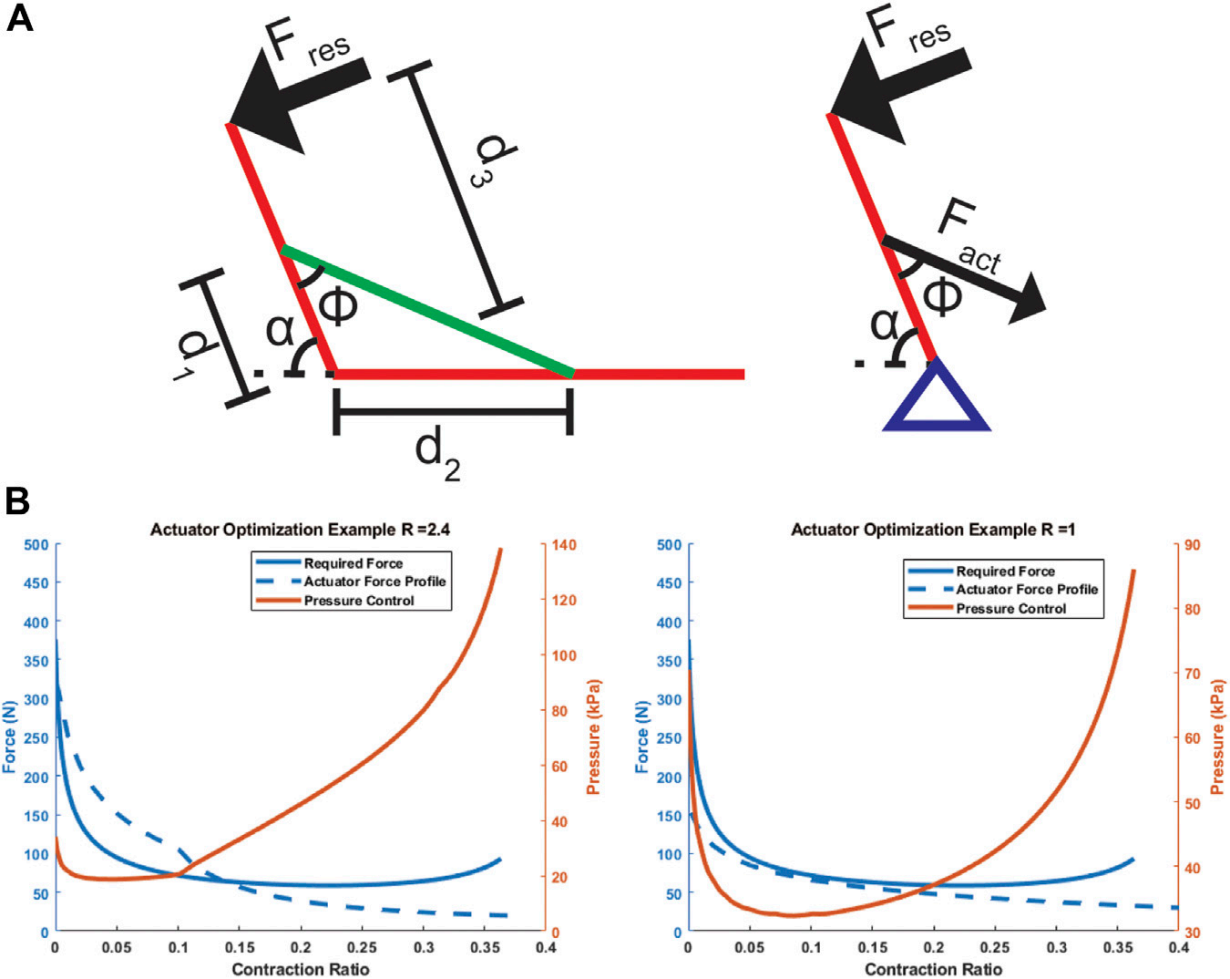
Schematic **(A)** and results **(B)** of actuator design selection example. **(A)** depicts a simplified arm setup with the actuator in green acting as a contractile muscle. **(B)** Depicts the desired force curve from a quasi-static analysis of the system, a force output curve for the chosen actuator, and a pressure control curve to match the actuator output to the desired force.

The example in Figure 11 demonstrates how the proposed model allows users to rapidly identify ideal actuator parameters (R) given requirements of their problem, and how they might use the calculated FCP to extract more questions about implementation. The second analysis with an actuator of R = 1 in Figure 11B illustrates this point, as that actuator requires a higher initial pressure and lower final pressure compared to the large pressure ranges required for the R = 2.4 case. However, the R = 1 actuator is not restricted to 36% contraction, and so would require a more complex sensing and control scheme to remain within the parameters of the problem. This demonstrates that the model’s calculated FCP can help in the consideration of other design parameters such as available pressure ranges, pressure control resolution, and required control scheme.

### Generalizing Model to Other Actuator Designs

As initially explained, this model assumes a thin inextensible film being pulled toward a skeleton or boundary with a vacuum. It is ideally applied to actuators such as the bellows and FOAMs as shown above, with additional components to account for the material of the skin and the system’s restoring force. From these two examples, we have demonstrated the model’s ability to be applied to different actuator geometries and its modularity in combining with external models. With these conclusions in mind, the model can be applied to other more mechanically complex actuator designs, and we list some examples in this section.

The vacuum powered curling actuator in Tawk et al., 2018 has the necessary thin skin and skeleton components, though it does not undergo linear contraction. Supplementary Figure S7 shows how the skin and boundary functions can be applied, with the boundary function changing during contraction to accommodate for the contractile cell’s bending. The output linear force can be converted to a torque by assuming a point load at the half-way height or other more complex force distributions along the height. A cantilever-based torsional spring model would also be overlaid to account for the restoring force of the bending skeleton.

Another possible application would be the buckling actuators in Yang et al., 2016; Supplementary Figure S7). The key issue in this case is the skin is a proportionally thick elastomeric layer, which may still allow for the inextensible assumption, but not that of a thin-film. A virtual thickness must be included in the model to account for the limitation on the stroke length, and a more complex spring model based on buckling would be included to model the skin’s restoring force. In that case, parameters such as skin thickness and material would be interesting to modulate, in addition to the usual contractile cell length and aspect ratio.

A process such as this–defining the skin and skeleton profile functions, reassessing the base model’s assumptions, and defining additional restoring force or loss components to overlay into model–can be repeated for other actuators that fall under the vacuum powered skin-skeleton category of actuators.

As a final note on further generalizability-the power of the virtual work principle is the computational simplicity in reaching an accurate estimate of a vacuum actuator’s force-contraction profile. This implies a possible approach to actuator design for more complex FCPs by controlling the volume loss rate. Actuators with origami skin patterns (Martinez et al., 2012; Li et al., 2017; Lee and Rodrigue, 2019), for example, have high potential in this application, as a new model could be developed and use the geometrical predictability of origami to generate new actuator designs to fit desired force-profiles.

### Limitations

In addition to illustrating the concept of virtual work, the curve in Figure 5 also illustrates one of the limitations of the model: the large initial force. This large force is due to the assumption that the skin at zero contraction is in full tension. In any practical implementation of an actuator, this does not hold for multiple reasons. First, there is often a small tolerance between the skin and the actuator skeleton, or in the case of the bellows actuator where there is no gap, asymmetries in the construction can lead to sections of the skin that are not in perfect tension. Perhaps more importantly, this assumption conflicts with the quasi-static nature of the system, as the pressure applies a radially-oriented (perpendicular) force on the skin that can only be compensated for by the tension on the skin (Li et al., 2017), requiring some initial curvature to allow for a radial component of the tension force. The effect of this physical inaccuracy is that, when comparing with experimental data, it is important to shift the model horizontally toward the negative x direction by some small percentage to eliminate the high peak force. After implementing a scaling factor that considers the above logic about a required initial skin curvature (Supplementary Section 1.1), the initial peak force was still higher than the experimental case, most probably due to the other flaws of fabrication that lead to an initial curvature in the skin. Given these unquantifiable factors, a shifting factor of 2% was implemented. This means in all comparisons of the model FCP with experimental data, the model FCP is shifted horizontally so its zero-point begins at a contraction of 2%.

In Figure 7, for the bellows with an R of 2, the model overpredicts the output force most probably due to this tension phenomenon. The large unsupported length of skin may have experienced higher cumulative pressure force and larger tension force compared to R = 0.5, 1, (as supported by the higher peak force for R = 2 in Figure 6A). This means the initial sagging of the skin for R = 2 may be greater than for 0.5 and 1, calling for a larger shift. Shifting the model by 2% for R = 2 eliminates the larger error in the estimated initial force and better approximates the early phase of the force profile.

Finally, in Figure 8, there is a consistent over-estimation of the initial force. We hypothesize that this is a result of the tension phenomenon combined with the fact that the skin in FOAMs, unlike for bellows actuators, is not directly anchored to the skeleton, implying that the sections of skin between the skeleton are slightly longer than the ideal perfect-tension model predicts. Due to the overall similarity in the profile shape, there is a better agreement with experimental results if the model is further shifted horizontally in this case as well.

## Conclusion

In this work, we hypothesized that the nonlinear force-contraction profile of a skin-skeleton vacuum actuator can be derived purely from a geometrical calculation of its volume loss rate. We tested this with the model and experiments presented here and proved that our hypothesis was valid. Though the magnitudes of the resultant forces are dependent on external properties, such as skin material, skeleton and skin restoring forces, and actuation pressure, we show that the shape of the FCP is dominated by the work done through volume loss rate. By applying the piston-scaled force and scaling factor and overlaying external models to capture the system’s restoring force, we can closely estimate the output of different actuators with much less computational and set-up time than developing actuator-specific FEM or analytical models. Importantly, these external models can be separately super-imposed on the FCP calculated by the virtual work model, preserving its modularity. Inaccuracies in this model are compensated for by its generalizability and utility as a design guiding tool to allow rapid parameter space sweeps. We demonstrate the latter point through a simple optimization example for a hypothetical application.

Future work would include developing a more robust system of modules that can model the restoring forces for various skin and skeleton geometries to interface with the virtual work model, as this is currently one of the largest sources of discrepancy between the model and experimental data. More work can also be done on understanding the effect of the skin material on the output force magnitude.

Notwithstanding these challenges, the generalized and modular nature of this framework enables its implementation as a design tool for a wide variety of vacuum actuators, provided they can be represented by one or more simplified skin-skeleton contraction cells. This ability to rapidly model a variety of actuators and actuator geometrical parameters has broad implications in improving design efficiency and speed in the fields of medical devices, robotics, and soft machines. For example, it can enable actuator design for application-specific force profiles, such as in patient-specific devices or robotic design, and the rapid computation time can be useful for dynamic closed-loop control applications in soft robotics.

## Data Availability

The raw data supporting the conclusions of this article will be made available by the authors, without undue reservation.
