# Supplementary material for: A Modular Geometrical Framework for Modelling the Force-Contraction Profile of Vacuum-Powered Soft Actuators
Source: Front Robot AI. 2021 Mar 3;8:606938. doi: 10.3389/frobt.2021.606938 (PMC7983108; doi:10.3389/frobt.2021.606938)
Supplement: Supplementary file 1 [file datasheet1.docx]

Supplementary Material

# Supplementary Data

A summary of supplementary figures referenced in the text:

Figure S1 is a picture of the experimental setup of the actuators in the mechanical tester, referenced in the methods section. Figure S6 shows the result of the compression test done for the FOAMs skeleton used in our experimental results; the spring constant k was extracted from this data and used in the model’s restoring force. Figure S7 is referred to in the section 4.4 to exemplify how the model may be used in other more complex actuator designs. Figures S8 and S9 serve to compare our model’s output with that of Felt et al. (2018) and Li et al. (2017).

## Derivation of scaling factor for predicting output force for actuators with different skin materials

To derive a theoretically-based method for the calculation of the scaling factor, we performed a set of FEM experiments using a bellows actuator setup, varying skin stiffness and thickness. From this data, we extracted a relationship between Young’s Modulus and thickness and the scaling factor which is based on Roark’s formula applied to a cable in tension.

Bellows FEM experiment setup

**Figure S2** depicts the FEM setup for the bellows actuators. S4R shell elements were used to model the skin, and a linear elastic material property was applied with varying Young’s Moduli in the range of 10-500MPa with a Poisson’s ratio of 0.3. The section thickness was varied from 0.02 to 0.35mm. To simulate the rings, displacement constraints were applied to node pairs radially around the skin at desired gaps, restricting their motion to be strictly along the actuator’s long axis. The system was given a total of three rings (two contractile cells). A rectangular prism shape (square cross-section of 20x20) was used for the actuator, as it allowed more consistent meshing and faster solution times than a circular cross section actuator – both were compared, and output force profiles were virtually identical given the same cross-sectional area. A pressure of -5kPa was applied to the actuators and an explicit simulation was run with a two-step loading process (similar to the method described for the FEM of the FOAM actuator in the main text). First, the nodes at the ends of the skin were fixed as the pressure was linearly increased until reaching the -5kPa, then the pressure was kept constant as a displacement condition was applied to one side of the actuator, allowing it to contract in a quasi-static state. The reaction forces at one of the ends were extracted and used as the FCP for that actuator.

Results from FEM and Scaling process

**Figure S3** shows the results of the FEM for varying stiffnesses (A) and thickness (B), as well as the trends in the maximum output forces for varying stiffness (C) and thickness (D). Finally **Figure S3E** shows the direct relationship between maximum output force and the cubed root of (E*t) – the next section expands on a theoretical reason for this.

To derive the function for scaling factor based on the material properties, we first extracted the FCP from the FEM models and scaled the output force by the force of an equivalent piston (2N). We then divided the maximum scaled force for each FEM experiment with the maximum force predicted by our unscaled virtual work model, to find the scaling factor for each E-t pairing. After finding that the trend between $\left( E*t \right)^{1/3}$ and the scaling factor was linear as in **Figure S3(E)**, we applied a linear fit to the FEM data to solve for our scaling factor equation: $s=0.1992*\left( Et \right)^{\frac{1}{3}}+0.0067$, where s is the scaling factor to be multiplied with the virtual work model to estimate the force output for given skin properties, E is the Young’s Modulus of the skin in MPa, and t is the thickness of the skin in mm.

Theoretical backing (Roark’s Formula)

The schematic in **Figure S4** shows the layout for Roark’s formula applied to a flexible cable, as described in ##Young & Budynas (2002), where $y_{max}$ is the skin’s sagging depth, $T$ is the tension on the skin equivalent to the output force, $w$ is a distributed load in N/m equivalent to the internal pressure, $E$ is the skin material’s Young’s Modulus, and $A$ is the cross-sectional area of the skin (not of the actuator), and, $L$ is the gap between rings. The equation is applied to the maximum force output of the actuators as it is valid for small deflections, $y_{max}$, of a cable with no initial sag, which in our FEM models corresponds to the initial zero-contraction point.

By plugging in the equation for $y_{max}$into the equation for $T$, one finds the following:

$$T=\frac{\left( wL \right)^{2/3}}{8*3^{\frac{1}{3}}}\left( 64EA \right)^{\frac{1}{3}}$$

Assuming the cross-sectional area of the skin to be $A=t*P_{act}$, where $t$ is the skin thickness and $P_{act}$ is the perimeter of the actuator’s cross section, we find that

$$\boldsymbol{T\sim}\left( \boldsymbol{E*t} \right)^{\frac{\boldsymbol{1}}{\boldsymbol{3}}}$$

This is corroborated by the FEM results in our scaling analysis above. Note there are other terms in the expression for T, but they are all incorporated in our model either as geometrical parameters or in the non-dimensional piston-scaled force, so we neglect them from the relationship for the scaling factor. The purpose of the scaling factor is to accommodate for aspects of the construction of an actuator that were not addressed by the virtual work model, namely the thickness and stiffness of the skin, which are known to be relevant and application-specific. As Roark’s formula for a cable shows, E and t are the only missing application-specific components that are not incorporated in the model.

## Bellows Restoring Force Experiment Methodology

In the main text, we hypothesize that one of the reasons for the discrepancy between our model and the experimental results for the bellows actuators is that the actuator’s skin imparts a restoring force especially towards the end of the contraction, where our model diverges most from the experiment. To test this hypothesis and quantify the significance of the bellows actuator’s restoring force, we performed an experiment on a set of four bellows actuators with an R of 1 (the same actuators used in the experiments whose results are in Figure 7).

First, one contractile cell of the actuator was clamped on a mechanical tensile tester (Instron 5944), meaning the first and second rings were clamped, while the last was left loose. The cell was held at a slight tension, a vacuum of -15kPa was applied to the actuator, and the tester was slowly lowered, allowing the cell to compress, until the output force was equal to zero, signaling the end of the contraction. The displacement equal to the end of contraction was recorded. The actuator’s contractile cell was then brought back to its original tensile position, the vacuum was turned off and the actuator was open to atmosphere. Again the tensile tester was slowly lowered, compressing the cell, and stopping when it had reached full contraction (when the force rose above 5N due to contact between the rings), and the force-displacement curve for this compression was extracted. This force profile is the actuator’s nonlinear restoring force.

The restoring force was then subtracted from the FCP for a bellows actuator of R=1 calculated by the virtual work model. This was done separately for each actuator as shown in **Figure S5**, given the varying contraction distances and restoring force curves. The model-estimated end-contraction points (where the plot crosses zero force) were extracted and compared with the measured end-contraction points, with an average error of 14%. This shows a significant improvement in the model’s ability to estimate the FCP of these actuators and supports the hypothesis that neglecting the loss due to the skin’s restoring force was a source of disagreement between our idealized model and the experimental measurements in Figure 6. As mentioned, the mechanics behind this nonlinear restoring force are complex and it is not within the scope of this work to develop an analytical model to derive it.

## Varying Skin Profile Functions

To explore the relevance of different skin profile functions, we performed a simple modelling run, where three different conic sections were used as the skin profile for a bellows actuator of R = 1: a parabola (as implemented in the main body of the paper), a hyperbola, and a half-ellipse with its vertices at the interface with the rings. The force-contraction profile for each was calculated and they are compared in **Figure S10.** Generally, there were no major differences between different profiles, though the parabola and hyperbola FCP follow each other virtually identically, while the ellipse FCP experiences a slightly less steep profile. This is likely because the ellipse experiences a more gradual drop in sag depth during contraction, whereas the hyperbola and parabola both lower more quickly. Overall, this shows the different conic sections similarly approximate a simple skin profile. Other more complex functions, such as the volume minimizing skin profile function in Felt *et al.* 2017, may lead to a more noticeable difference, despite following the same trend. More complex skin geometries, such as for an origami-based actuator, would require much more complex skin profile functions.

# Supplementary Figures and Tables

## Supplementary Figures


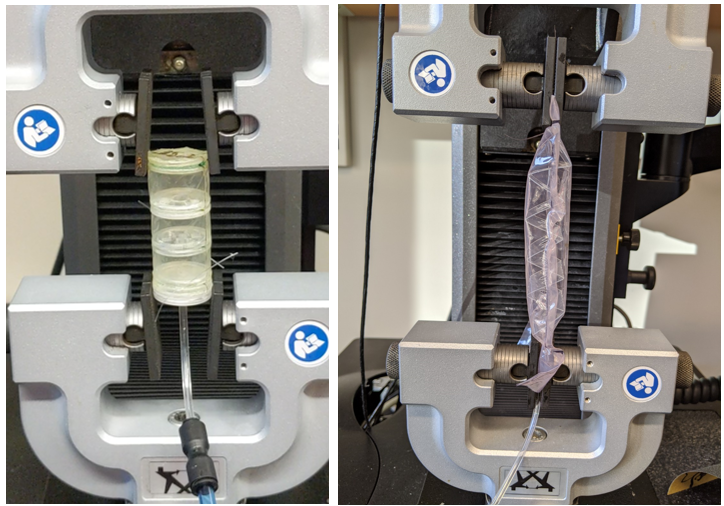


**Supplementary Figure 1.** Experimental Setup for bellows (left) and FOAMs (right)


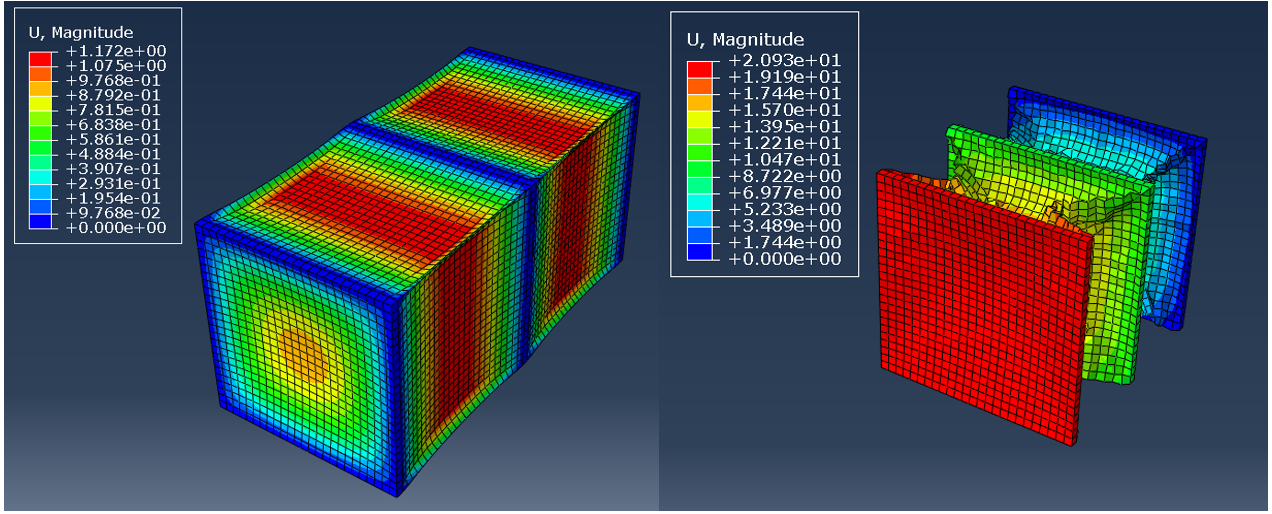


**Supplementary Figure 2.** Results of bellows FEM setup for extracting FCP, at zero and 50% contraction. R = 1 , E = 237MPa and t = 0.1mm. Displacement, U, in mm.


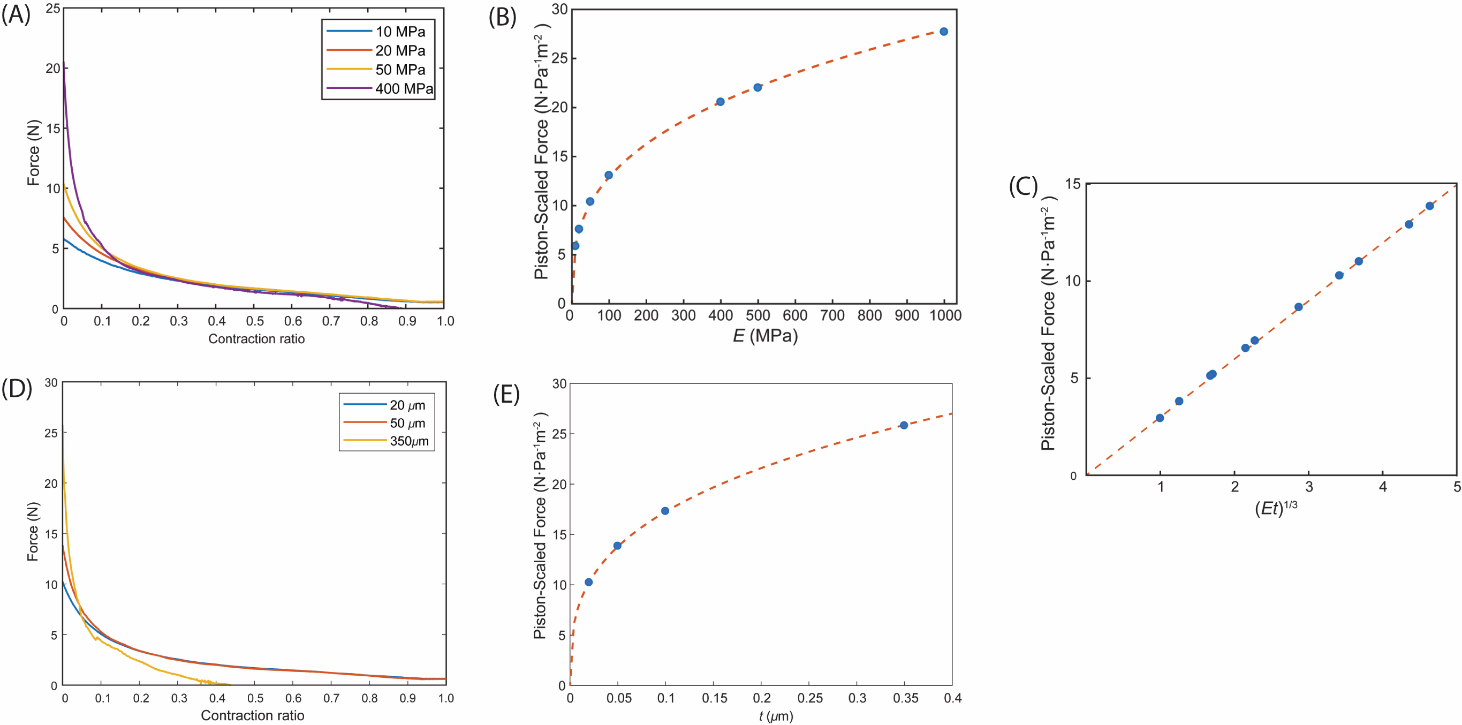


**Supplementary Figure 3.** Actuator skin material scaling factor for virtual work model in relation to skin tension stiffness given identical actuator dimensions. (*Et)^(1/3)* is an expression of skin material tension stiffness, where *E* is Young’s modulus and *t* is the thickness of the skin material used in the actuator.


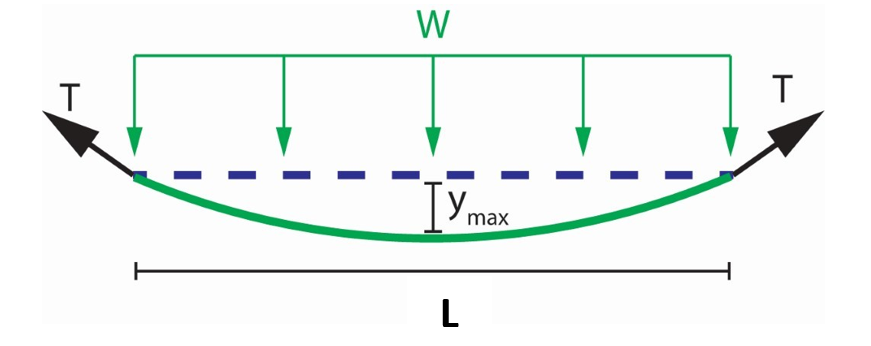


**Supplementary Figure 4.** Schematic of cable under uniform load for Roark’s formula.


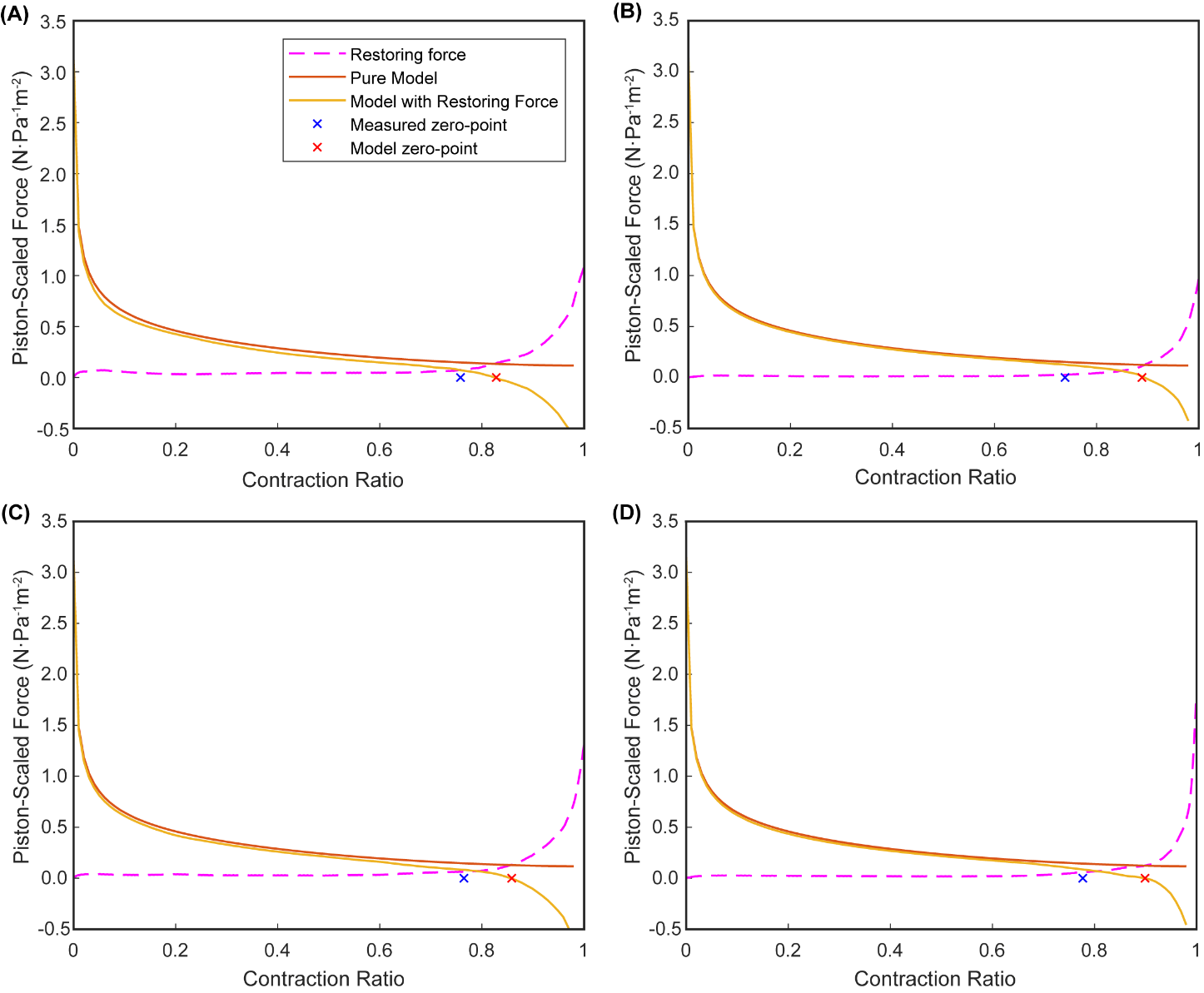


**Supplementary Figure 5.** Compression testing results with an average of 14% error between measured and modelled end of contraction. (A)-(D) are the four replicates for bellows actuators for R=1


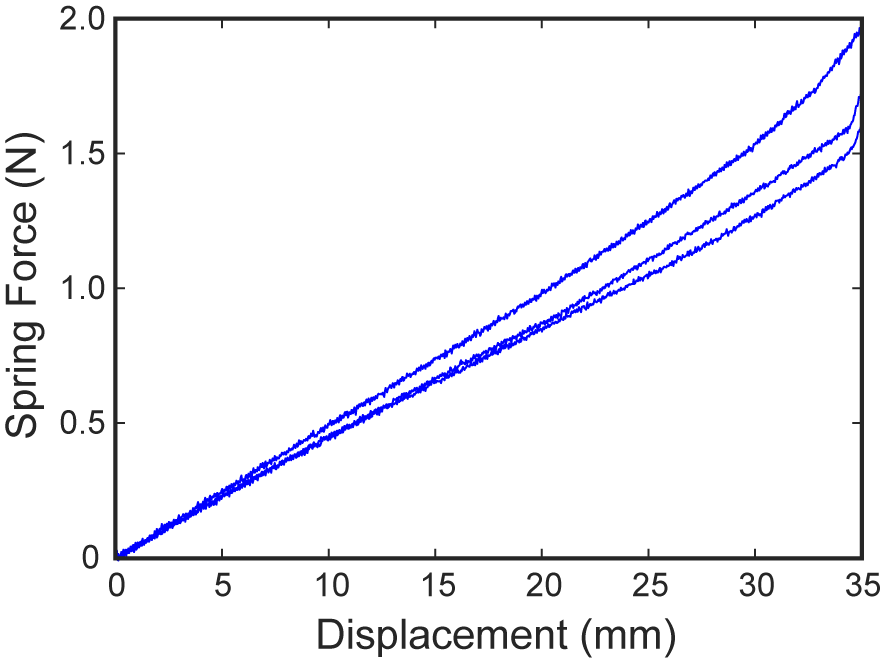


**Supplementary Figure 6.** Measured spring force from 30-degree zigzag skeletons during a compression test (n=3) as described in the Methods section.


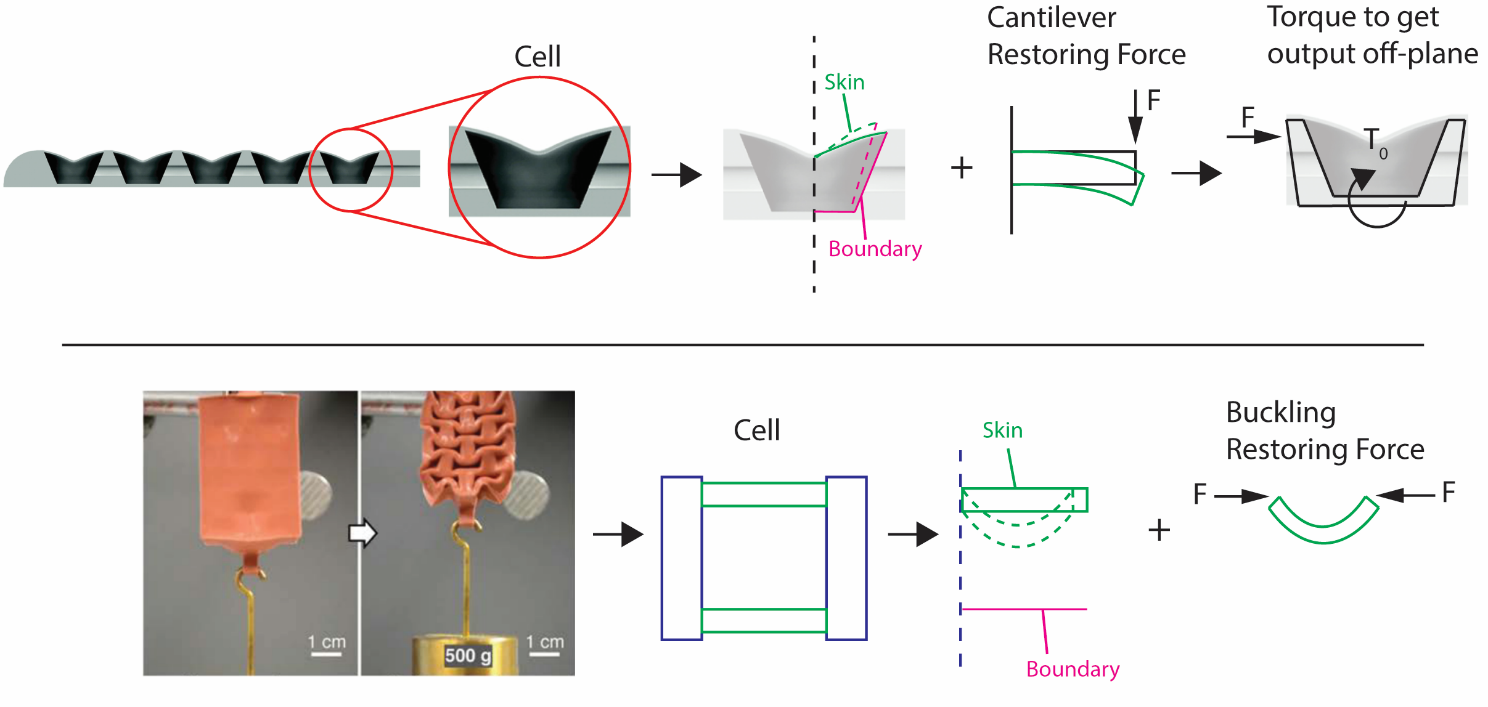
**Supplementary Figure 7.** Schematic of how our model may be applied to different more complex actuators, such as a curling actuator (Tawk et al. 2018) and a buckling elastomeric actuator (Yang et al. 2016).


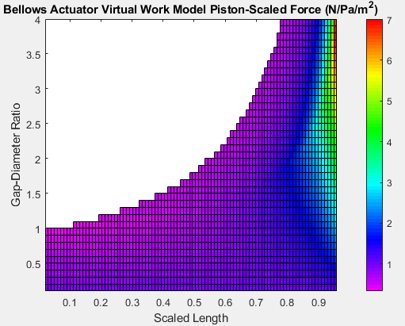

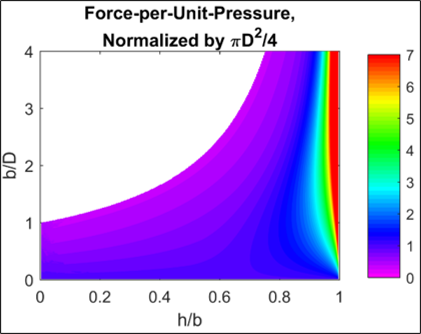


**Supplementary Figure 8.** Comparison of our parameter sweep (Left) with Felt et al. 2018 (Right).


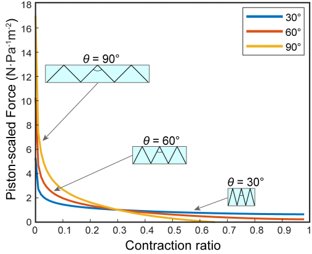


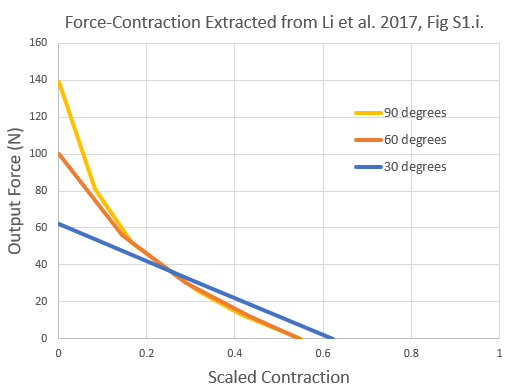

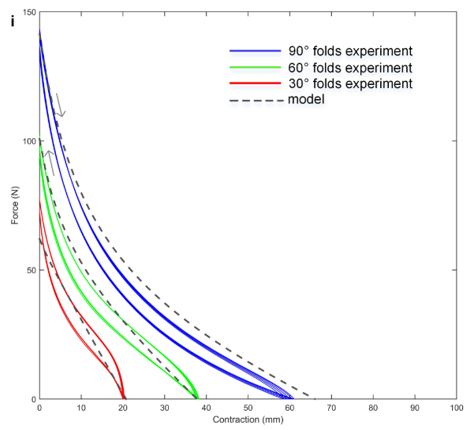


**Supplementary Figure 9.** Comparison of our FOAM model (Top) without inclusion of the spring force with Li et al. (2017) (Bottom-Right) with the inclusion of the skeleton spring force to match the experiment. A scaled version of the Li et al. data is shown (Bottom-Left) to allow comparison of trends between our model and Li et al.


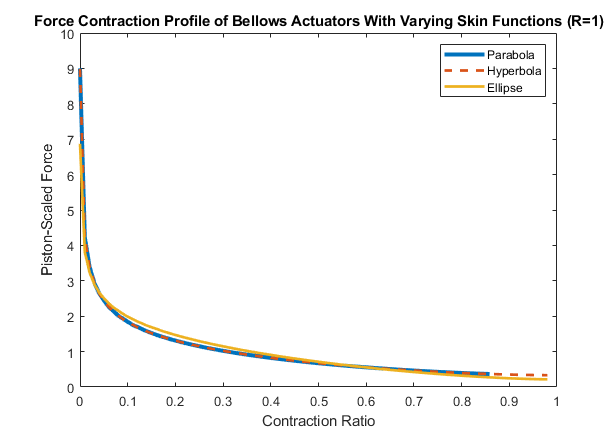


**Supplementary Figure 10**. Force contraction profile for bellows actuators with varying skin profile functions for *R* = 1
